# Role of the IL-1 Pathway in Dopaminergic Neurodegeneration and Decreased Voluntary Movement

**DOI:** 10.1007/s12035-016-9988-x

**Published:** 2016-06-29

**Authors:** Andrea Stojakovic, Gilberto Paz-Filho, Mauricio Arcos-Burgos, Julio Licinio, Ma-Li Wong, Claudio A. Mastronardi

**Affiliations:** 1 Department of Clinical and Experimental Medicine, Division of Cell Biology, Faculty of Medicine and Health Sciences, Linköping University, Linköping, Sweden; 2 The Arcos-Burgos Group, Genome Science Department, John Curtin School of Medical Research, Australian National University, Canberra, Australia; 3 Mind and Brain Theme, South Australian Health and Medical Research Institute and Flinders, University of South Australia, Adelaide, Australia

**Keywords:** Open-field, Rotarod, Substantia nigra, Dopaminergic neuron, Microglia

## Abstract

Interleukin-1 (IL-1), a proinflammatory cytokine synthesized and released by activated microglia, can cause dopaminergic neurodegeneration leading to Parkinson’s disease (PD). However, it is uncertain whether IL-1 can act directly, or by exacerbating the harmful actions of other brain insults. To ascertain the role of the IL-1 pathway on dopaminergic neurodegeneration and motor skills during aging, we compared mice with impaired [caspase-1 knockout (casp1^−/−^)] or overactivated IL-1 activity [IL-1 receptor antagonist knockout (IL-1ra^−/−^)] to wild-type (wt) mice at young and middle age. Their motor skills were evaluated by the open-field and rotarod tests, and quantification of their dopamine neurons and activated microglia within the substantia nigra were performed by immunohistochemistry. IL-1ra^−/−^ mice showed an age-related decline in motor skills, a reduced number of dopamine neurons, and an increase in activated microglia when compared to wt or casp1^−/−^ mice. Casp1^−/−^ mice had similar changes in motor skills and dopamine neurons, but fewer activated microglia cells than wt mice. Our results suggest that the overactivated IL-1 pathway occurring in IL-1ra^−/−^ mice in the absence of inflammatory interventions (e.g., intracerebral injections performed in animal models of PD) increased activated microglia, decreased the number of dopaminergic neurons, and reduced their motor skills. Decreased IL-1 activity in casp1^−/−^ mice did not yield clear protective effects when compared with wt mice. In summary, in the absence of overt brain insults, chronic activation of the IL-1 pathway may promote pathological aspects of PD per se, but its impairment does not appear to yield advantages over wt mice.

## Introduction

A growing body of evidence suggests that inflammatory mediators play a pivotal role in aging and neurodegenerative disorders such as Parkinson’s disease (PD), a condition that affect more than 1 % of the population aged 60 years and older in industrialized countries [1]. A hallmark for the pathophysiology of PD is dopaminergic neurodegeneration within the substantia nigra (SN) pars compacta (SNpc), which leads to decreased striatal dopamine (DA) levels and affects the control of voluntary movements [2]. Activation of microglia, either by age or different insults, has been proposed as a key neuroinflammatory process leading to the progression of dopaminergic neurodegeneration [3]. Activated microglia release a number of inflammatory factors, which in turn trigger a neuroinflammatory cascade causing neuronal damage [4, 5].

Interleukin-1 (IL-1) is one of the most well-known proinflammatory cytokines that act within the brain during different insults and neurodegenerative diseases, including PD [6]. The IL-1 system involves two essential agonists, IL-1 alpha (IL-1α) and IL-1 beta (IL-1β), as well as the endogenous antagonist, IL-1 receptor antagonist (IL-1ra) [7]. Both IL-1α/β exert similar biological effects by binding to IL-1 receptor 1 (IL-1R1), whereas IL-1ra blocks IL-1α/β biological activity by competing with them by binding to IL-1R1 [7, 8]. Upon binding to IL-1R1, IL-1ra does not trigger any second messenger signal [7, 8]. Another key component of the IL-1 system is caspase-1 (casp1), a cysteine protease that, when cleaved, activates the immature form of IL-1. The activation of casp1 controls the rate-limiting step in the conversion of pro-IL-1beta into the mature biologically active cytokine. Thus, IL-1ra and casp1 are two key targets that modulate the IL-1 system.

The crucial roles of impairing or activating the IL-1 pathway have been evidenced by the diametrically opposed response that caspase-1 knockout (casp1^−/−^) and interleukin-1 receptor antagonist knockout (IL-1ra^−/−^) mice, respectively, display after being challenged with high doses of lipopolysaccharide from Gram-negative bacteria (LPS) [9, 10]. In fact, casp1^−/−^ mice are largely resilient [9], whereas IL-1ra^−/−^ mice are much more susceptible to death after receiving high doses of LPS [10]. Within the central nervous system (CNS), casp1^−/−^ mice display a lower inflammatory response after receiving a systemic challenge with LPS when compared to wild-type (wt) mice [11, 12]. In contrast, IL-1ra^−/−^ mice display an enhanced CNS inflammatory response and are more sensitive to a central inflammatory challenge [13]. Moreover, the latter study showed that activated microglia play an essential role in the neuroinflammatory cascade [13].

Within the brain, IL-1 is mainly synthesized and released by activated microglia [14]. Several lines of research, including preclinical [15–17] and post-mortem studies [18], have suggested that IL-1 pathway activation is involved in mechanisms causing neurotoxicity [15–18]. It is not completely understood whether the activation of IL-1 causes neurotoxicity per se or if its involvement in dopaminergic neurodegeneration requires preexistent triggering insults. There is evidence contradicting the direct role of IL-1β on neuronal death [19, 20], but supporting its role in exacerbating brain damage caused by other triggering factors such as traumatic, ischemic, or excitotoxic stimuli [20].

In order to better understand the role of the IL-1 pathway in neurotoxicity and in the dysregulation of the nigrostriatal system during aging, we employed a noninvasive approach to study locomotion and motor coordination activity in young adult mice with impaired (casp1^−/−^) or overactive (IL-1ra^−/−^) IL-1 pathway. We tested the hypothesis that these strains display differential locomotion and body balance regulation during aging. At the end of the study, we also estimated the number of dopaminergic neurons and activated microglia within the SN.

## Materials and Methods

All animal experiments were performed according to the rules and regulations of the “Australian code of practice for the care and use of animals for scientific purposes.” All experimental protocols were approved by the Australian National University Animal Experimentation Ethics Committee (AEEC).

### Animals

Groups of wt, casp1^−/−^ [9], and IL-1ra^−/−^ [10] mice, bred in C57BL/6 background, were evaluated. All mice (10–12 per group) were fed a regular chow diet (food and water ad libitum), housed in groups of up to five animals per cage and maintained under standard living conditions (22 ± 2 °C, 12-/12-h light/dark cycle) for the entire duration of the studies.

### Behavioral Tests

Locomotion, coordination, and balance skills were evaluated when mice were young (42–45 days old), and 9 months later (i.e., when they became over 10 months old). This period was selected based on a previous study that showed that immunological challenged mice with monthly intraperitoneal (i.p.) injections of LPS showed impaired motor skills after 9 months of receiving the first injection [21]. Locomotion was determined by placing each individual mouse in an open-field arena (48 cm × 48 cm), and recording its activity for 32 min with a video camera placed from the top of the arena. The software “Viewer 3” (Biobserve Gmbh, St. Augustin, Germany) was used for data collection and processing, which provided measures of the total distance travelled.

Coordination and balance skills were ascertained by employing a rotarod apparatus (Panlab, Harvard apparatus, Barcelona, Spain). Prior to starting data collection, mice were trained for 4–6 days and were given three trials per day in order to achieve maximal performance in the rotarod test [22–24]. All of the mice were included since they were successfully trained for this behavioral paradigm, and they looked healthy throughout the entire duration of the study as per routine inspections of their fur, body weight, response to handling, and home cage social behavior. The mice were frequently monitored by the researchers and technical staff of the Australian National University animal facility. Upon placing the mice on the rotating drum, the initial speed was 4 revolutions per minute (rpm), and it was accelerated to 40 rpm within 2 min. Mice were given three trials with 2-min breaks between trials. The latency to fall from the rotating drum was measured in seconds. Mice that showed better balance and coordination skills were able to remain for a longer period of time on the rotating drum. The final result was calculated as the mean of the latency to fall from the rotarod apparatus during the three trials. In the infrequently observed event in which the mouse had not fallen from the rotating drum after 2 min, it was removed from the apparatus and returned to its home cage [21]. Behavioral assessments in the rotarod and open-field tests are frequently used to determine parkinsonism-related outcomes [25–28].

Henceforth, the first behavioral assessments obtained at young age (42–45 days old) will be referred as “baseline,” and those obtained 9 months later as “9 months.” Thus, for simplicity purpose, baseline is considered as the “zero point” and any other mentioned experimental time-point will reflect the time elapsed from baseline unless otherwise stated.

### Immunohistochemistry of Dopamine Neurons and Microglia Cells

#### Tissue Preparation

Fifteen months after the initiation of the experiments, mice were anesthetized with an intraperitoneal injection of ketamine/xylazine (100/10 mg/kg, adjusted to a volume of 0.1 ml/10 g of body weight) and transcardially perfused with a solution of cold phosphate-buffered saline (PBS) and heparin (5 U/ml) within 3 min. The mouse brain was removed and frozen in chilled isopentanol and stored at −80 °C until use. Brains were sliced using a cryostat (set at −12 °C) in 15-μm coronal sections throughout the entire SN.

#### Immunohistochemistry

We followed previously reported protocols for tyrosine hydroxylase (TH) and microglia staining with minor modifications [29, 30]. Brain sections were collected on gelatin-coated slides, air-dried and post-fixed in 4 % paraformaldehyde (pH∼7.4 in PBS) for 8–10 min. After a PBS wash (three times within 5 min), sections were incubated for 20 min in 3 % peroxide mixed in methanol in order to inactivate the endogenous peroxidase. Sections were incubated with 5 % normal goat serum and 0.015 % Triton X-100 in PBS. After 30 min of incubation, the sections were incubated with rabbit anti-TH antibody (1:1000 dilution; Life Technology, cat# P21962) [29], or rat anti-mouse CD68 monoclonal antibody (1:700; AbD Serotec, cat# MCA341GA) in 0.015 % Triton X-100 for 24 h at 4 °C [30]. On the following day, the slides were washed in PBS and incubated with biotinylated goat anti-rabbit (1:200; Vector Laboratories, cat# PK-6101) or goat anti-rat secondary antibody (1:500; Vector Laboratories, cat# BA-9400) for 1 h. After a PBS wash, slides were reacted with avidin-biotin complex (ABC; Vector Laboratories, cat# PK-6101) for 30 min. In the final step, slides were washed with PBS and incubated with 3,3′-diaminobenzidine (DAB; Sigma, cat# D3939) and 30 % peroxide until color development (5–7 min). Slides were counterstained with hematoxylin (30 s), dehydrated in alcohol gradient (80, 90, 100, 100 %), immersed in xylene, mounted with DPX slide mounting medium (Sigma, cat# 05622), and coverslipped. All the incubations were done at room temperature, unless mentioned otherwise.

#### Quantification of Dopamine Neurons and Microglia

In order to assess the loss of dopamine neurons and the number of activated of microglia cells, two adjacent series of eight consecutive slides (15 μm of thickness) were collected to sample the region of SN (rostral to caudal: −2.65 to −3.61 mm posterior to bregma) [21]. Thus, the two series of eight evenly spaced slides were obtained every 90 μm and were used for counting dopamine neurons or activated microglia cells. The borders of SN were defined in order to exclude the ventral tegmental area (VTA) from counting [31]. The area defined for counting covered the entire area from the rostral part of SNpc to the caudal end of SN pars reticulata (SNpr), excluding those TH neurons that interspersed with oculomotor nerve rootlet. Images were obtained with an IX2-UCB Olympus digital camera (Olympus, Tokyo, Japan) and analyzed manually using the ImageJ software (http://imagej.nih.gov from NIH). Positive CD68 microglia cells in SN were counted manually on a Nikon Eclipse 50i microscope (Nikon, Tokyo, Japan). The picture magnifications were ×20 and ×40 for microglia and ×20 for TH.

### Statistical Analysis

Data were analyzed with GraphPad software version 5 (GraphPad Software, La Jolla California USA) and expressed as mean ± standard error of the mean (S.E.M). One-way ANOVA followed by Newman-Keuls post hoc test was used to analyze the differences among the three genotypes (wt, casp1^−/−^, and IL-1ra^−/−^). Values of *P* < 0.05 were considered significant.

## Results

### Age-Related Decline of Motor Skills in IL-1ra^−/−^ Mice

**Fig. 1:**
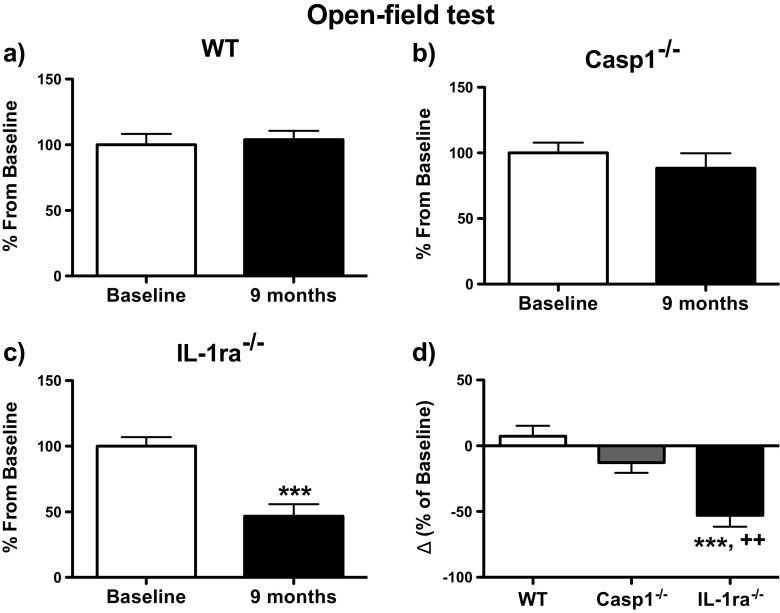
Total distance travelled during open-field test. **a**–**c** Comparison of the total distance travelled by wt (*n* = 10), casp1^−/−^ (*n* = 8) and IL-1ra^−/−^ (*n* = 12) mice between baseline and 9 months. Baseline performance was considered as 100 %. The total distance at 9 months is expressed as percentage of baseline. **d** Comparison of the average differences between 9 months and baseline for each genotype; the differences were calculated for each mouse and expressed as percentage of baseline by using the following formula: [(Td 9M − Td B) / Td B] × 100, where “Td 9M” and “Td B” represent the total distance at 9 months and baseline, respectively. In this and in following graphs, *each column* represents the mean, and the *bar above* denotes the standard error of the mean (SEM). For the statistical comparison between two groups (**a**–**c**), we employed paired *t* tests, whereas for the comparison of three groups (**d**), we performed one-way ANOVA followed by Newman-Keuls post hoc test. ***P < 0.001 vs. wt; ^++^ *P* < 0.01 vs. casp^−/−^

In order to better understand the role of IL-1 signaling on dysregulation of the nigrostriatal system, the open-field and the rotarod tests were performed to assess locomotion and coordination/balance in all groups of mice. We evaluated their performance at young age (baseline) and at 9 months later (i.e., when they became nearly/or 10.5 months old). Figure 1a–c displays the percentage of change in total distance travelled in the open-field arena between baseline and 9 months by mice of the three genotypes. Results were expressed as percentage change relative to baseline measurements, which was considered to be 100 %. This was calculated as follows: total distance at 9 months / total distance traveled at baseline × 100 %. IL-1ra^−/−^ mice showed a significant 53 % decline in locomotor activity (*P* < 0.0001), whereas wt and casp1^−/−^ displayed similar activity at both time-points (Fig. 1a–c). Figure 1d shows the comparison of change of distance traveled from baseline and 9 months later between the three genotypes. At 9 months, the locomotor activity was reduced by 53 % in IL-1ra^−/−^ mice when compared to baseline, and this decline was significantly different from the changes observed in wt (*P* < 0.001) and casp1^−/−^ mice (*P* < 0.01) (Fig. 1d).

**Fig. 2:**
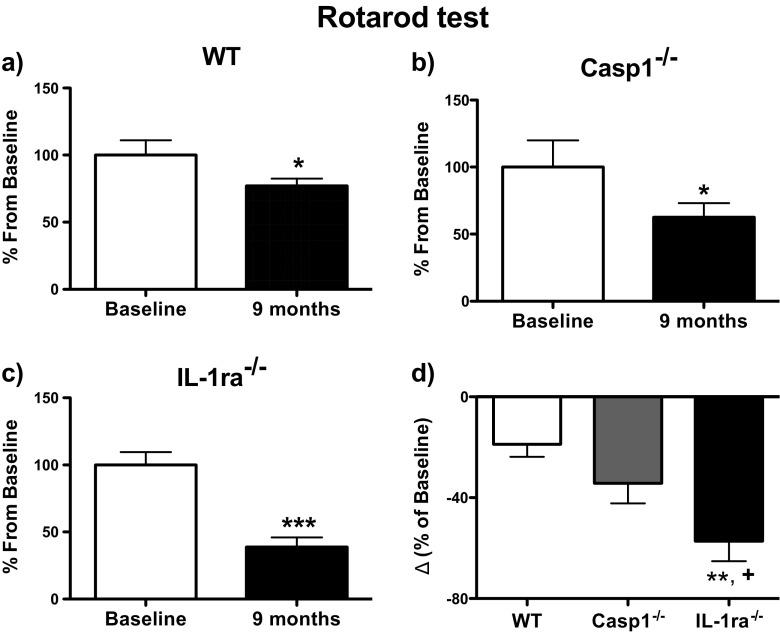
Rotarod performance. **a**–**c** Comparison of the average latency to fall from the rotating drum displayed by wt (*n* = 10), casp1^−/−^ (*n* = 8), and IL-1ra^−/−^ (*n* = 12) mice at baseline and 9 months. The differences were calculated for each mouse and expressed as percentage of baseline by using the following formula: [(L9M − LB) / LB] × 100, where “L9M” and “LB” represent the latency to fall at 9 months and baseline, respectively. **d** Comparison of the average differences between 9 months and baseline for each genotype; the differences were calculated for each mouse and expressed as percentage of baseline by using the following formula: [(L9M − LB) / LB] × 100. For the statistical comparison between two groups (**a**–**c**), we employed paired *t* tests, whereas for the comparison of three groups (**d**), we performed one-way ANOVA followed by Newman-Keuls post hoc test. ***P < 0.001 vs. wt; **P < 0.01 vs. wt; *P < 0.05 vs. wt; ^+^ *P* < 0.05 vs. casp1^−/−^

The rotarod apparatus is largely used to test coordination and balance skills in rodents where the latency to fall from the rotating drum is recorded and used as a measure of their motor coordination and balance abilities [32, 33]. Figure 2a–c displays the change between baseline and 9 months later in the average elapsed time to fall down from the rotating drum of the rotarod apparatus for all groups of mice. The latency to fall from the rotating drum obtained after 9 months of the first assessments was expressed as percentage relative to baseline, which was considered to be 100 % (Fig. 2a–c). Significant levels of decline in their performance of 23 % (*P*<0.05), 37 % (*P*<0.05), and 63 % (*P*<0.001) were observed in the wt, casp1^−/−^, and IL-1ra^−/−^ groups, respectively (Fig. 2a–c). The genotype comparison showed that the rotarod performance of IL-1ra^−/−^ mice was significantly more pronounced than the 23 % decline observed in wt (*P* < 0.01) and the 37 % decline displayed by casp1^−/−^ mice (*P* < 0.05) (Fig. 2d).

### Role of IL-1 Signaling in Microglia Activation and Neurodegeneration of Dopamine Neurons in SN

**Fig. 3:**
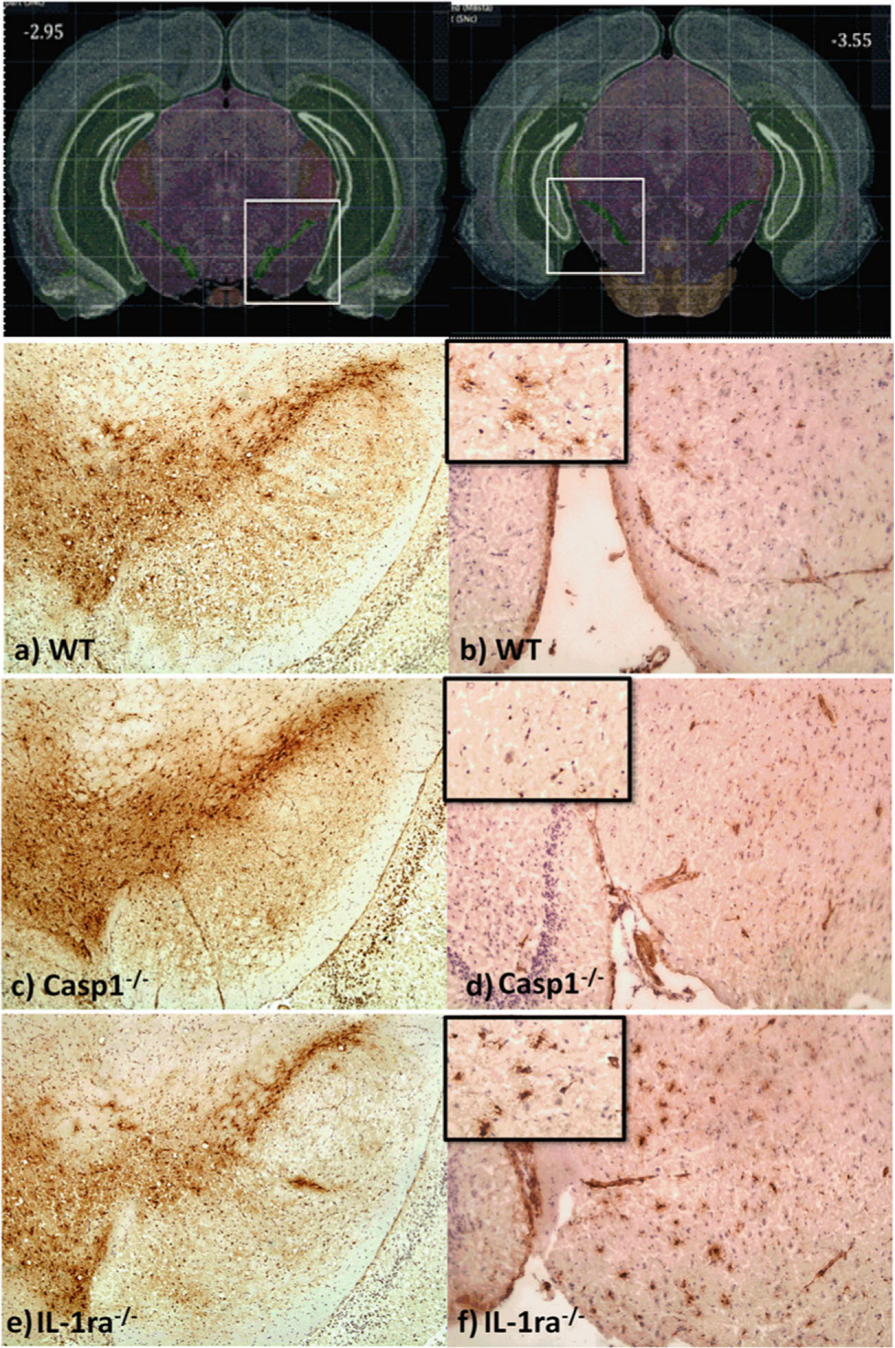
Immunostaining of TH-positive neurons and CD68-positive microglia cells. Frozen sections of mouse brains were stained with anti-TH and anti-CD68 antibody at 15 months. Positive TH immunostaining of **a** wt, **c** casp1^−/−^, and **e** IL-1ra^−/−^, and positive CD68-immunostaining of **b** wt, **d** casp1^−/−^, and **f** IL-1ra^−/−^ mice

**Fig. 4:**
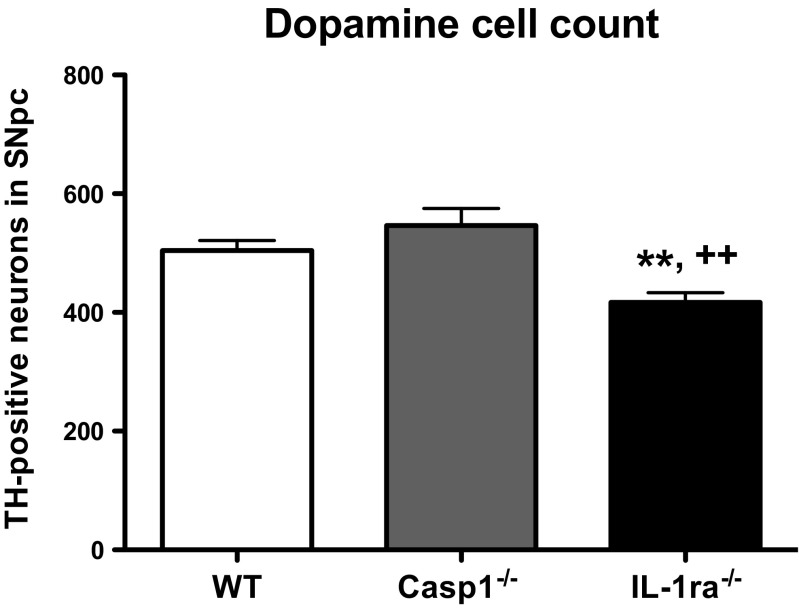
Dopamine neurons cell count. Mice brains were stained with anti-TH at 15 months. The total number of TH-positive cells from eight evenly spaced frozen sections that encompassed the entire SN of wt (*n* = 11), casp1^−/−^ (*n* = 7), and IL-1ra^−/−^ (*n* = 7) mice was evaluated from the unilateral side of SNpc. Data were analyzed by one-way ANOVA (Newman-Keuls post hoc test). *Error bars* represent standard errors. **P < 0.01 vs. wt; ^++^ *P* < 0.01 vs. casp1^−/−^

In order to assess the role of IL-1 on neuronal degeneration of dopamine neurons, the total number of TH-positive neurons was quantified from eight evenly spaced frozen sections of the brain that encompassed the entire region of the SNpc. Representative pictures displaying positive TH staining of dopamine neurons in SNpc of wt, casp1^−/−^, and IL-1ra^−/−^ mice are shown in Fig. 3 (left panels). As presented in Fig. 4, there was a reduction of up to 24 % of dopamine neurons in IL-1ra^−/−^ mice in comparison to wt (*P* < 0.05) and casp1^−/−^ (*P* < 0.001) mice.

**Fig. 5:**
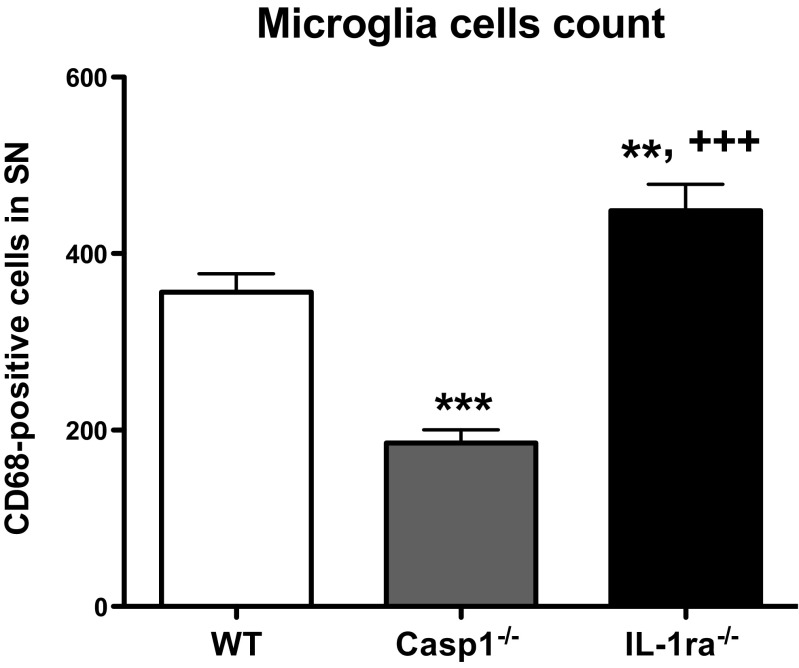
Microglia cell count. Eight evenly spaced frozen sections that encompassed the entire SN were stained with anti-CD68, a marker for activated microglia. Total count of CD68-positive cells was counted unilaterally in the region of SN of wt (*n* = 11), casp1^−/−^ (*n* = 6), and IL-1ra^−/−^ (*n* = 6) mice and analyzed by one-way ANOVA followed by Newman-Keuls post hoc test. *Error bars* represent standard errors. **P < 0.01 vs. wt; ***P < 0.001 vs. wt; ^+++^ *P* < 0.0001 vs. casp1^−/−^

To further understand the implication of activated microglial cells in the neurodegeneration of dopamine neurons, activated microglia/macrophages were stained with the CD68 marker and counted in the area of the SNpc and SNpr. A positive CD68 staining of activated microglia cells in the SN of wt, casp1^−/−^, and IL-1ra^−/−^ is presented in Fig. 3 (right panels). As presented in Fig. 5, casp1^−/−^ mice had a significantly lower number of activated microglia (approximately 50 %) in comparison to wt and IL-1ra^−/−^ mice, suggesting that these mice show an overall lower inflammation within the brain. On the other hand, IL-1ra^−/−^ mice had significant higher number of activated microglia in comparison to the other two genotypes.

## Discussion

We have ascertained the role of the IL-1 pathway on long-term locomotion and motor coordination in wt, IL-1ra^−/−^, and casp1^−/−^ mice displaying normal, overactive, or impaired IL-1 pathway, respectively. Our hypothesis was that, in the absence of overt brain insults, chronic activation of the IL-1 system evokes parkinsonism-related behaviors during aging, whereas impairment of IL-1 activity in casp1^−/−^ mice results in neuroprotection and better performance. Our results in IL-1ra^−/−^ mice suggest that overactivation of microglia cells by IL-1 decreases the number of dopaminergic neurons, which in turn reduces locomotion and motor coordination to promote pathological aspects of parkinsonism. However, our results show that deficiency of casp1 does not exert a clear protection in sparing motor skills during aging, despite the fact that these mice display fewer activated microglia.

In our study, we tested mice in an open-field arena to assess their voluntary locomotor activity and in an accelerating rotarod apparatus to determine their motor coordination and balance. These two behavioral paradigms are widely used to evaluate parkinsonism-related behavior in rodents [25–28]. Baseline was defined as the time-point we started the experiments with young adult mice (42–45 days old), whereas 9 months was the time we finalized their behavioral assessments (i.e., mice were over 10 months old). This period of time was selected based on previous data showing that endotoxin-challenged mice with five monthly intraperitoneal LPS injections displayed decreased motor skills capabilities 9 months after the first injection [21].

IL-1ra^−/−^ mice displayed a much more pronounced decrease in both motor coordination and locomotor activity when compared to wt and casp1^−/−^ mice 9 months later. The latter suggests that chronic exacerbation of the IL-1 pathway affects dopaminergic neurotransmission and impairs motor skills. This concept is supported by our histological studies because IL-1ra^−/−^ mice displayed an increased number of activated microglia cells and a decreased number of dopaminergic neurons compared to wt and casp1^−/−^ mice. The pathophysiological role of microglial-induced neuroinflammation in PD was originally described by McGeer et al. in 1988, when they showed the presence of activated microglia in the SN of PD patients [18, 34]. Studies carried in other species such as monkeys [35] and rodents [36–38] also provided strong evidence of activated microglia in impaired dopaminergic neurotransmission [35–38]. Furthermore, the prominent role of IL-1β has been described in rats that were centrally injected with adenovirus expressing IL-1β, which caused neurotoxicity within hippocampus and SN [15–17, 39], and in IL-1-deficient mice that were injected with LPS [38]. It is noteworthy that the chronic expression of IL-1β within the SN in rats was sufficient to elicit microglial activation and dopaminergic neurodegeneration [16], which were exacerbated by concomitant systemic expression of IL-1β [39]. The data described about IL-1ra^−/−^ mice herein are aligned with the latter mentioned studies and support the concept that IL-1-induced neuroinflammation could have resulted in activation of the neuroinflammatory cascade causing a decreased number of TH neurons within the SN [15, 16, 39]. Hence, decreased dopaminergic cell bodies in the SN could explain the lower capabilities in motor skills displayed by IL-1ra^−/−^ mice 9 months after performing the first open field and rotarod tests.

However, there appears to be some controversy surrounding the putative direct neurotoxic role of IL-1 within the brain. For instance, it has been also suggested that some of the previously provided in vivo evidence [15–17] could have been influenced by confounding technical variables (i.e., inflammation caused by intracerebral injection, viral administration, or nonphysiologic doses of IL-1β) [40]. Moreover, it has been proposed that IL-1β cannot exert neurotoxicity per se within the CNS but rather exacerbates the inflammatory cascade elicited by other insults [40]. That concept is supported by mouse studies showing that chronic overexpression of human IL-1β for a period of 2 months within the hippocampus does not seem to cause evident signs of overt neurodegeneration [41]. Additionally, in vitro studies also suggested that IL-1β cannot exert neurotoxicity per se [42, 43]. Since our approach did not require central interventions that could cause brain inflammation, it is quite likely that increased IL-1 is largely responsible for the impairment in motor skills that IL-1ra^−/−^ mice showed 9 months later of starting the behavioral experiments. Since we employed a traditional constitutive knockout model that lacks IL-1ra, our results are influenced by the combined action of both IL-1β and IL-1α. Therefore, it is likely that some of the seemingly differences between our data and other studies reporting the lack of action of IL-1β in brain neurotoxicity could be, at least partially, due to the fact that in this mouse model, we tested the unopposed actions to both IL-1β and IL-1α. Additionally, our findings might be affected not only by the overactivation of the IL-1 system within the CNS but also in the periphery, which collectively could affect dopaminergic neurotransmission [6, 39].

As mentioned above, the putative protective impact of decreased activation of the IL-1 pathway was ascertained in casp1^−/−^ mice, since this strain lacks biologically active IL-1β and shows much lower levels of IL-1α than wt mice [9]. Furthermore, in previous studies, we have shown that casp1^−/−^ displayed a lower level of brain inflammation than wt mice after administering systemic LPS [11, 44]. However, both casp1^−/−^ and wt mice displayed nonsignificant differences in their locomotor activity and motor coordination performance. It is noteworthy that casp1^−/−^ mice showed a lower number of activated microglia that did not result in a higher number of TH neurons, and/or better motor capabilities than wt mice.

Identifying key players driving the neuroinflammatory cascade during PD could aid in the development of novel therapeutic strategies to counter some of its deleterious effects. Successful novel interventions in preclinical studies continue showing the potential benefit of targeting the neuroinflammatory cascade to protect dopaminergic neurodegeneration in rodent models of PD [45, 46]. Clinical and postmortem studies have also demonstrated the occurrence of CNS inflammation in living and deceased subjects with PD, respectively [34, 47]. Furthermore, epidemiological data suggested that the chronic use of anti-inflammatory drugs decreases the risk of developing PD [48]. Consequently, targeting neuroinflammatory mediators in patients with PD has been proposed as a potential clinical approach to halting dopaminergic neurodegeneration [49, 50]. Our current results in IL-1ra^−/−^ mice (the strain displaying activation of the IL-1 system) and other studies previously reported in rodents support the concept that IL-1 is involved in microglia activation leading to increased dopaminergic neurodegeneration [15–17]. Thus, therapeutic strategies antagonizing IL-1 might yield a potential benefit in treating patients with PD.

There are some potential limitations to our study that should be acknowledged. The behavioral data compared motor skills assessments obtained at baseline and 9 months, whereas the histological studies were performed at 15 months. The elapsed time between these experiments could have favored the progression of dopaminergic neurodegeneration. In female mice receiving five monthly LPS injections, the loss of dopamine neurons reached 37 % 9 months after the first LPS injection and progressed to 55 % after 20 months of the first endotoxin administration [21]. In our studies, dopaminergic neurodegeneration may also have progressed between 9 and 15 months. Since some of the mice of the current study also served as controls for another project that required ascertaining insulin resistance by performing intraperitoneal glucose tolerance test (GTT), we carried out the histological studies at a later time-point to reduce the number of mice employed in our experiments. The ascertaining of GTT involved an acute exposure to a moderate level of stress. We cannot rule out that the GTT studies could have also affected the histological experiments. In order to minimize this putative confounding effect, mice were euthanized shortly after the test was done (7–10 days later). Mice deficient in IL-1ra bred in the BALB/c background spontaneously developed polyarthropathy, which closely resembles rheumatoid arthritis in humans [51]. The IL-1ra^−/−^ mice employed here were bred in the C57Bl6 background, which do not show similar signs of chronic inflammation [51]. However, the IL-1ra^−/−^ mice used in our studies are approximately 20 % smaller than wt mice, which could have influenced some of the data reported here. Also, it was recently shown that casp1-targeted mice carry a 129 ES cell-derived mutation in the *casp11* gene, which appeared to be involved, at least in part, in their resilience to LPS-induced lethality [52]. Thus, we cannot rule out that some of the results observed in casp1^−/−^ are also influenced by the lack of casp11 activity.

Future studies should be carried out to address the putative contribution of brain overactivation of the IL system. One possibility to address this issue would be to design experiments employing mice with brain-specific deficiency of IL-1ra and perform similar measurements for histological and behavioral assessments. Data from those experiments could be compared with the results reported presently.

Collectively, our data suggest that long-term exposure to increased IL-1β/IL-1α could evoke parkinsonism-related outcomes such as impairment in motor skills, a higher number of activated microglia, and reduced number of TH-neurons as described in IL-1ra^−/−^ mice. Since all of these changes occurred during normal aging of the IL-1ra^−/−^ mice in the absence of brain inflammatory insults that are frequently caused by intracerebral injections in animal models of PD, we provide novel evidence suggesting that chronic activation of the IL-1 system can elicit parkinsonism per se. In contrast, long-term exposure to reduced levels of IL-1β/IL-1α as observed in casp1^−/−^ mice failed to alter long-term motor skills despite the fact that these mice showed a lower number of activated microglia when compared to wt mice. The elucidation of the inflammatory mediators that play key roles in PD is crucial to develop new therapeutic strategies to overcome this deleterious condition.

## Abbreviations

ABC: Avidin-biotin complex
AEEC: Australian National University Animal Experimentation Ethics Committee
casp1: Caspase-1
casp1^−/−^: Caspase-1 knockout
CNS: Central nervous system
DA: Dopamine
DAB: 3,3′-Diaminobenzidine
GTT: Intraperitoneal glucose tolerance test
i.p.: Intraperitoneal
IL-1: Interleukin-1
IL-1R1: IL-1 receptor 1
IL-1ra^−/−^: IL-1 receptor antagonist knockout
IL-1β: IL-1 beta
IL-1α: IL-1 alpha
LPS: Lipopolysaccharide
PBS: Phosphate-buffered saline
PD: Parkinson’s disease
rpm: Revolutions per minute
SEM: Standard error of the mean
SN: Substantia nigra
SNpc: Substantia nigra pars compacta
SNpr: Substantia nigra pars reticulata
TH: Tyrosine hydroxylase
VTA: Ventral tegmental area
WT: Wild type

## References

[1] Nussbaum RL, Ellis CE (2003). Alzheimer’s Disease and Parkinson’s Disease. N Engl J Med.

[2] Dauer W, Przedborski S (2003). Parkinson’s disease: mechanisms and models. Neuron.

[3] Block ML, Hong JS (2005). Microglia and inflammation-mediated neurodegeneration: multiple triggers with a common mechanism. Prog Neurobiol.

[4] Godbout JP (2005). Exaggerated neuroinflammation and sickness behavior in aged mice following activation of the peripheral innate immune system. FASEB J.

[5] Dilger RN, Johnson RW (2008). Aging, microglial cell priming, and the discordant central inflammatory response to signals from the peripheral immune system. J Leukoc Biol.

[6] Collins LM (2012). Contributions of central and systemic inflammation to the pathophysiology of Parkinson’s disease. Neuropharmacology.

[7] Dinarello CA (2011). A clinical perspective of IL-1β as the gatekeeper of inflammation. Eur J Immunol.

[8] Kondo S (1995). Interleukin-1 receptor antagonist suppresses contact hypersensitivity. J Invest Dermatol.

[9] Li P (1995). Mice deficient in IL-1 beta-converting enzyme are defective in production of mature IL-1 beta and resistant to endotoxic shock. Cell.

[10] Hirsch E (1996). Functions of interleukin 1 receptor antagonist in gene knockout and overproducing mice. Proc Natl Acad Sci U S A.

[11] Mastronardi C (2007). Caspase 1 deficiency reduces inflammation-induced brain transcription. Proc Natl Acad Sci U S A.

[12] Kamens J (1995). Identification and characterization of ICH-2, a novel member of the interleukin-1 beta-converting enzyme family of cysteine proteases. J Biol Chem.

[13] Craft JM (2005). Interleukin 1 receptor antagonist knockout mice show enhanced microglial activation and neuronal damage induced by intracerebroventricular infusion of human beta-amyloid. J Neuroinflammation.

[14] Herx LM, Rivest S, Yong VW (2000). Central nervous system-initiated inflammation and neurotrophism in trauma: IL-1 beta is required for the production of ciliary neurotrophic factor. J Immunol.

[15] Depino A (2005). Differential effects of interleukin-1beta on neurotoxicity, cytokine induction and glial reaction in specific brain regions. J Neuroimmunol.

[16] Ferrari CC (2006). Progressive neurodegeneration and motor disabilities induced by chronic expression of IL-1beta in the substantia nigra. Neurobiol Dis.

[17] Carvey PM (2005). Intra-parenchymal injection of tumor necrosis factor-alpha and interleukin 1-beta produces dopamine neuron loss in the rat. J Neural Transm.

[18] McGeer PL, Itagaki S, McGeer EG (1988). Expression of the histocompatibility glycoprotein HLA-DR in neurological disease. Acta Neuropathol.

[19] Lawrence CB, Allan SM, Rothwell NJ (1998). Interleukin-1beta and the interleukin-1 receptor antagonist act in the striatum to modify excitotoxic brain damage in the rat. Eur J Neurosci.

[20] Allan SM, Tyrrell PJ, Rothwell NJ (2005). Interleukin-1 and neuronal injury. Nat Rev Immunol.

[21] Liu Y (2008). Endotoxin induces a delayed loss of TH-IR neurons in substantia nigra and motor behavioral deficits. NeuroToxicology.

[22] Zhu BG (2012). Optimal dosages of fluoxetine in the treatment of hypoxic brain injury induced by 3-nitropropionic acid: implications for the adjunctive treatment of patients after acute ischemic stroke. CNS Neuroscience and Therapeutics.

[23] Yamada MH (2012). Impaired glycinergic synaptic transmission and enhanced inflammatory pain in mice with reduced expression of vesicular GABA transporter (VGAT). Mol Pharmacol.

[24] Kreutzfeldt M (2013). Neuroprotective intervention by interferon-γ blockade prevents CD8+ T cell–mediated dendrite and synapse loss. J Exp Med.

[25] Kelly MA (1998). Locomotor activity in D2 dopamine receptor-deficient mice is determined by gene dosage, genetic background, and developmental adaptations. J Neurosci.

[26] Vijitruth R (2006). Cyclooxygenase-2 mediates microglial activation and secondary dopaminergic cell death in the mouse MPTP model of Parkinson’s disease. J Neuroinflammation.

[27] Abdelsalam RM, Safar MM (2015). Neuroprotective effects of vildagliptin in rat rotenone Parkinson’s disease model: role of RAGE-NFkB and Nrf2-antioxidant signaling pathways. J Neurochem.

[28] Liu W (2015). Neuroprotective effects of lixisenatide and liraglutide in the 1-methyl-4-phenyl-1,2,3,6-tetrahydropyridine mouse model of Parkinson’s disease. Neuroscience.

[29] Lee J-Y (2009). Cytosolic labile zinc accumulation in degenerating dopaminergic neurons of mouse brain after MPTP treatment. Brain Res.

[30] Yin F (2010). Exaggerated inflammation, impaired host defense, and neuropathology in progranulin-deficient mice. J Exp Med.

[31] McNeill TH, Koek LL (1990). Differential effects of advancing age on neurotransmitter cell loss in the substantia nigra and striatum of C57BL/6N mice. Brain Res.

[32] Deacon RM (2013) Measuring motor coordination in mice. J Vis Exp (75). doi: 10.3791/2609 10.3791/2609 PMC3724562 23748408

[33] Jones BJ, Roberts DJ (1968). The quantiative measurement of motor inco-ordination in naive mice using an acelerating rotarod. J Pharm Pharmacol.

[34] McGeer PL (1988). Reactive microglia are positive for HLA-DR in the substantia nigra of Parkinson’s and Alzheimer’s disease brains. Neurology.

[35] McGeer PL (2003). Presence of reactive microglia in monkey substantia nigra years after 1-methyl-4-phenyl-1,2,3,6-tetrahydropyridine administration. Ann Neurol.

[36] Marinova-Mutafchieva L (2009). Relationship between microglial activation and dopaminergic neuronal loss in the substantia nigra: a time course study in a 6-hydroxydopamine model of Parkinson’s disease. J Neurochem.

[37] Gao L (2015). Infiltration of circulating myeloid cells through CD95L contributes to neurodegeneration in mice. J Exp Med.

[38] Tanaka S (2013). Activation of microglia induces symptoms of Parkinson’s disease in wild-type, but not in IL-1 knockout mice. J Neuroinflammation.

[39] Pott Godoy MC, Ferrari CC, Pitossi FJ (2010). Nigral neurodegeneration triggered by striatal AdIL-1 administration can be exacerbated by systemic IL-1 expression. J Neuroimmunol.

[40] Shaftel SS, Griffin WS, O’Banion MK (2008). The role of interleukin-1 in neuroinflammation and Alzheimer disease: an evolving perspective. J Neuroinflammation.

[41] Shaftel SS (2007). Chronic interleukin-1beta expression in mouse brain leads to leukocyte infiltration and neutrophil-independent blood brain barrier permeability without overt neurodegeneration. J Neurosci.

[42] Hailer NP (2005). Interleukin-1beta exacerbates and interleukin-1 receptor antagonist attenuates neuronal injury and microglial activation after excitotoxic damage in organotypic hippocampal slice cultures. Eur J Neurosci.

[43] Rothwell N (2003). Interleukin-1 and neuronal injury: mechanisms, modification, and therapeutic potential. Brain Behav Immun.

[44] Mastronardi CA (2015). Temporal gene expression in the hippocampus and peripheral organs to endotoxin-induced systemic inflammatory response in caspase-1-deficient mice. Neuroimmunomodulation.

[45] Fu SP (2015). Anti-inflammatory effects of BHBA in both in vivo and in vitro Parkinson’s disease models are mediated by GPR109A-dependent mechanisms. J Neuroinflammation.

[46] Nassar NN (2015). Saxagliptin: a novel antiparkinsonian approach. Neuropharmacology.

[47] Politis M, Lindvall O (2012). Clinical application of stem cell therapy in Parkinson’s disease. BMC Med.

[48] Gagne JJ, Power MC (2010). Anti-inflammatory drugs and risk of Parkinson disease: a meta-analysis. Neurology.

[49] Tansey MG, Goldberg MS (2010). Neuroinflammation in Parkinson’s disease: its role in neuronal death and implications for therapeutic intervention. Neurobiol Dis.

[50] Luo L (2010). Ten years of Nature Reviews Neuroscience: insights from the highly cited. Nat Rev Neurosci.

[51] Horai R (2000). Development of chronic inflammatory arthropathy resembling rheumatoid arthritis in interleukin 1 receptor antagonist-deficient mice. J Exp Med.

[52] Kayagaki N (2011). Non-canonical inflammasome activation targets caspase-11. Nature.

